# Suggested Screening Criteria for Gardnerella vaginalis Based on Established Risk Factors

**DOI:** 10.7759/cureus.72036

**Published:** 2024-10-21

**Authors:** Afrida Sara, Apurva Ramanujam, Dhiya Ram, Kelley L Davis, Stephanie Nagy, Marc M Kesselman

**Affiliations:** 1 Rheumatology, Dr. Kiran C. Patel College of Osteopathic Medicine, Nova Southeastern University, Fort Lauderdale, USA; 2 Microbiology, Dr. Kiran C. Patel College of Allopathic Medicine, Nova Southeastern University, Fort Lauderdale, USA

**Keywords:** bacterial vaginosis, ethnicity, gardnerella vaginalis, haemophilus haemolyticus vaginalis, hygiene practices, risk factors, screening criteria, sexual practices, smoking, socioeconomic status

## Abstract

Vaginal infections in women occur due to the downregulation of lactobacilli and the upregulation of *Gardnerella vaginalis* (*GV*), leading to bacterial vaginosis (BV). While certain practices are recognized as risk factors for contracting *GV *infections, this scoping review highlights the severity and importance of other lesser-known risk factors, such as smoking, ethnicity, socioeconomic status (SES), genetics, and anatomy, which can be used to develop an updated point-based screening tool for clinicians. A total of 438 articles were gathered from Embase, Ovid MEDLINE, and Web of Science, and after screening, 31 articles were included. There was a positive association with the presence of *GV *in those who were sexually active, practiced sexual penetrative vaginal acts, had frequent vaginal and/or receptive oral sexual activity, had unprotected sex, and used insertive contraception (intrauterine device, vaginal rings, and condoms). Women with primary school education levels showed a higher *GV* colonization increase compared to those with secondary or university education levels, and girls from the highest SES reported the lowest incidence. *GV *was the predominant bacteria found among sub-Saharan, South African, African Surinamese, Ghanaian, Tanzanian, and Kenyan women. In the USA, self-identified “black” women had a higher prevalence of *GV *in their vaginal microbiome compared to self-identified “white” women; however, this was the opposite in pregnant women. Significant data show that nicotine use has a strong correlation with increased incidence of *GV*. Other factors that were found to be associated with *GV *infections were the increase of sialidase A gene in *GV*, short cervix (<25mm), and women who performed vaginal douching. Timely screening of *GV *is vital, especially in high-risk populations, such as pregnant and immunocompromised patients, who may present with more severe and exaggerated symptoms if they contract BV. This paper proposes a numerical scale for evaluating patients' likelihood of contracting a *GV *infection during their hospital visit.

## Introduction and background

The vaginal microbiota is partially responsible for maintaining the cervicovaginal environment that contributes to the state of reproductive health in women. Imbalances in the vaginal microbiota lead to diseased states of reproductive health such as bacterial vaginosis (BV). The optimal cervicovaginal microbiome is dominated by *Lactobacillus* bacteria, which confer protection against infections in the reproductive tract [1]. The most common vaginal infection in women is caused by the downregulation of lactobacilli and the upregulation of *Gardnerella*, which leads to BV [1,2]. *Gardnerella vaginalis (GV)* is a predominant anaerobic, gram-variable *Coccobacillus* bacterium currently accepted as the prevailing etiological agent of BV, with an occurrence rate ranging between 5% and 70% among countries [3]. Research has identified *GV* to be a component of normal vaginal flora in over 50% of asymptomatic women; however, *GV* has been determined to play a pivotal role in both asymptomatic and symptomatic BV [4]. Many cases of BV often go undiagnosed, as only 50% of affected women present with symptoms like vaginal discharge, odor, itching, and burning during urination. The virulence factors of GV, namely, hemolysin and mucus-degrading sialidases, allow the bacteria to produce biofilms and attach to epithelial cells, which displaces pre-coated lactobacilli and thus weakens protection against infection [1,2]. The overgrowth of *GV* as a result of the reduction of lactobacilli leads to a nonoptimal cervicovaginal environment that predisposes women to BV [1]. The prevalence rate highlights the importance of clarifying causative factors, screening guidelines, and access to treatment.

The long-term pregnancy-related complications of *GV* affect both the mother and the fetus, which include premature labor, premature rupture of membranes, chorioamnionitis, neonatal meningitis, postpartum endometriosis, and low birth weight. Despite these devastating adverse events, routine screening for BV during pregnancy is yet to be mandated, therefore making appropriate screening of at-risk individuals and timely treatment of symptomatic patients all the more imperative [5]. Complications outside of pregnancy include an increased risk of developing endometriosis, postsurgical infections, pelvic inflammatory disease, and increased susceptibility to contracting sexually transmitted infections, including HIV [5,6].

BV is diagnosed using Amsel criteria and the Nugent scoring system. An elevated pH level is recognized as the most sensitive but least specific criterion and is significantly associated with BV [7]. Differential diagnoses for BV include atrophic vaginitis, candidiasis, cervicitis, chlamydia, desquamative inflammatory vaginitis, gonorrhea, herpes simplex, and trichomoniasis, which can be ruled out by pelvic examination, speculum exam, wet mount, and cervical swab cultures [6,8]. Treatment is not necessary for asymptomatic patients, and 30% of BV cases resolve on their own; however, if the patient experiences distress (unfavorable social, emotional, and sexual impacts or economic strain), treatment regimens may be started, including oral and topical metronidazole and topical clindamycin [8]. 

While certain sexual practices are recognized as risk factors for contracting *GV* infections, this scoping review highlights the severity and importance of other lesser-known risk factors, which can be used to develop an updated point-based screening tool for clinicians. 

## Review

Methods

An initial literature search was conducted on Google Scholar to gain a brief overview of the topic, after which certain keywords were sought from relevant articles. As *GV* was the pathogenic organism in question, the keyword “Gardnerella vaginalis” was searched across abstracts, keywords, and titles, alongside second-level keywords, including “Corynebacterium vaginale” OR “corynebacterium vaginalis” OR “Haemophilus haemolyticus vaginalis” OR “Haemophilus vaginalis.” Next, Boolean terms “Gardnerella vaginalis” AND “risk factors” OR “Smoking” were used. Due to smoking being linked to numerous comorbidities, the term was searched across abstracts, titles, and keywords to explore its association, if any, with *GV*. Due to the review focusing specifically on risk factors, no search terms that singled out a population based on geographic region or age group, for example, were included. The search strategy was replicated across three databases, namely, Embase, Ovid MEDLINE, and Web of Science, which yielded 58 articles. Articles were excluded due to the following: publication date being outside the established timeframe for this review and articles being irrelevant to the specific research question. 

**Figure 1:**
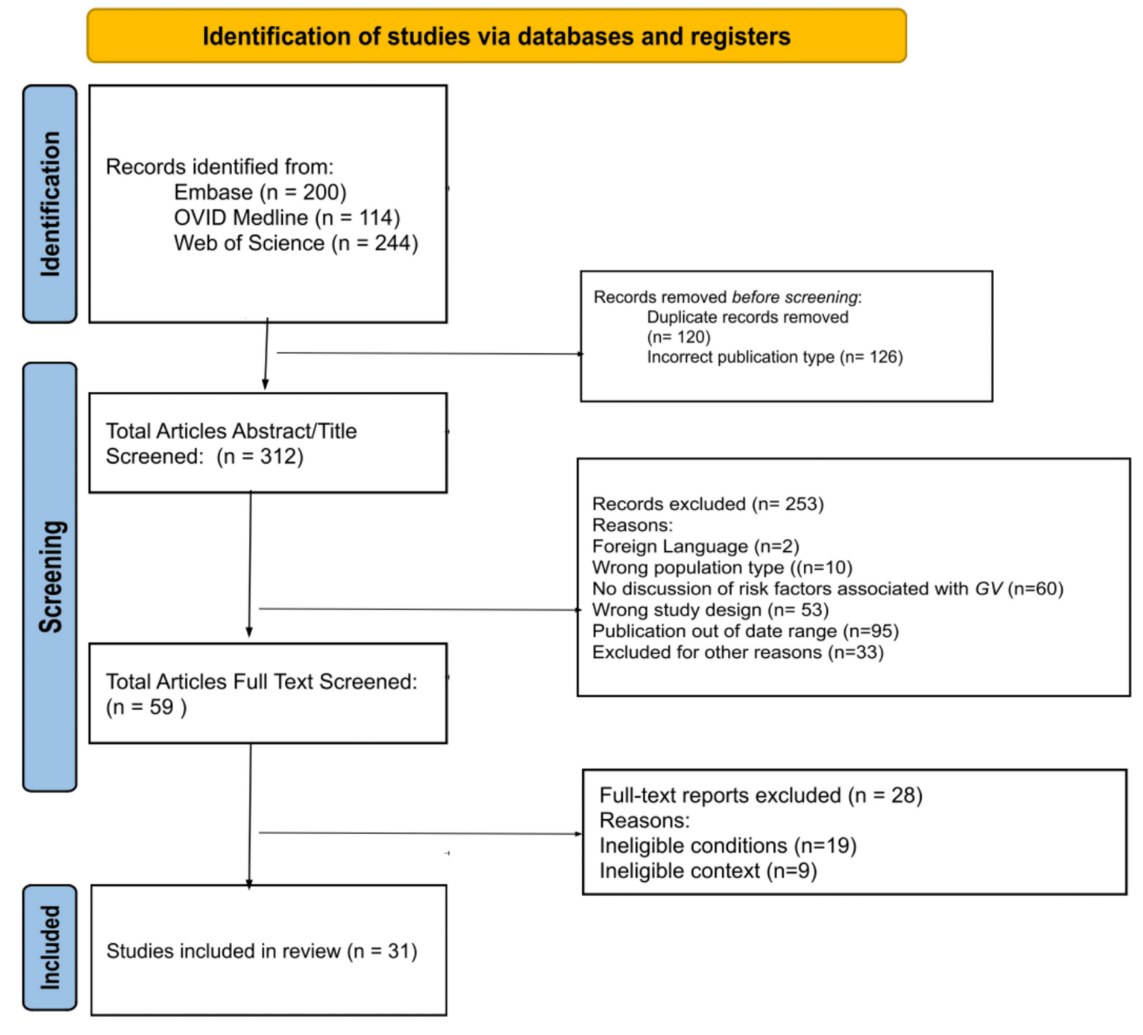
PRISMA flowchart of the selection procedure PRISMA: Preferred Reporting Items for Systematic Reviews and Meta-Analyses, GV: *Gardnerella vaginalis.* Source: [9].

Before the article screening and selection processes, duplicates were removed, leaving 438 articles to be analyzed. After the initial title and abstract screening, 379 articles were excluded, leaving 59 articles. Twenty-eight more articles were excluded after full-text screening, with 31 articles included in the final review. The Preferred Reporting Items for Systematic Reviews and Meta-Analyses (PRISMA) flowchart details the study’s article screening and selection processes (Figure 1) [9]. 

Results

Data were extrapolated from the final 31 papers and charted independently by each of the three reviewers. The variables utilized in the form included aspects of the study design, risk factor category, and number of subjects. 

Table 1 shows the 31 articles analyzed for the study design, risk factor category, and number of subjects in selected studies.

**Table 1:** Analysis of selected studies Source: [10-40].

Study	Study design	Risk factor category	Number of subjects
Beamer et al. [10]	Clinical trial	Sexual practices	440
Borgdorff et al. [11]	Cross-sectional study	Sexual practices, ethnicity	610
Datcu et al. [12]	Clinical trial	Sexual practices	196
Plummer et al. [13]	Longitudinal study	Sexual practices	100
Plummer et al. [14]	Longitudinal study	Sexual practices, smoking	100
Payne et al. [15]	Cohort study	Sexual practices, hygiene practices	1001
Jespers et al. [16]	Longitudinal study	Sexual practices	110
Jespers et al. [17]	Clinical trial	Sexual practices, ethnicity	430
Mitchell et al. [18]	Cohort study	Sexual practices	97
Mitchell et al. [19]	Cross-sectional study	Sexual practices	320
Fethers et al. [20]	Cohort study	Sexual practices, smoking	339
Balashov et al. [21]	Cohort Study	Sexual practices	60
Shihab et al. [22]	Interventional prospective study	Sexual practices	100
Achilles et al. [23]	Cohort study	Sexual practices	266
Huang et al. [24]	Cohort study	Sexual practices	120
Francis et al. [25]	Cohort study	Socioeconomic status, genetics	25
Machado et al. [26]	Cross-sectional study	Socioeconomic status, ethnicity	206
Desseauve et al. [27]	Cross-sectional study	Socioeconomic status, smoking	14,193
Wang et al. [28]	Clinical trial	Ethnicity	688
Lennard et al. [29]	Cohort study	Ethnicity	298
Balkus et al. [30]	Clinical trial	Ethnicity	234
Tossas et al. [31]	Cohort study	Ethnicity	4851
Fettweis et al. [32]	Cohort study	Ethnicity, smoking	1684
Muzny et al. [33]	Longitudinal study	Ethnicity	164
Salinas et al. [34]	Cross-sectional study	Ethnicity	95
Nelson et al. [35]	Cross-sectional study	Smoking	36
Tužil et al. [36]	Cross-sectional study	Smoking	250
Vallejo et al. [37]	Retrospective cohort study	Smoking	4752
Santos-Greatti et al. [38]	Cross-sectional study	Smoking	526
Ugur et al. [39]	Cross-sectional study	Smoking	114
Silvano et al. [40]	Cohort study	Anatomy	97
Total			32502

After analyzing the findings of the articles, commonalities were found regarding sexual practices, SES, ethnicity, smoking, the virulence of *GV*, anatomic variation, and hygiene practices.

Sexual Practices 

Data extracted from several studies point to sexual intercourse as a risk factor for *GV* infection, due to alterations in the cervicovaginal *Lactobacillus*-to-*GV* ratio introduced through sexual practices [10-14]. Single, nonvirgin women are more likely to be infected by *GV* than women who have not had sexual intercourse [15]. Studies done on women aged 18-22 and girls aged 14-19 demonstrate a significant increase in the occurrence of *GV* in those who have experienced sexual debut compared to those who have not engaged in sexual intercourse [16-18]. In terms of sexual intercourse, penetrative vaginal acts aside from penile-vaginal sex, such as digital-vaginal, have also demonstrated an increased risk for *GV* [19]. Additionally, having more than one sexual partner over a three-month period, greater than 10 lifetime sexual partners, and frequent vaginal and/or receptive oral sexual activity were shown to be positively associated with the presence of *GV* [16,17,20,21]. 

Furthermore, the lack of contraception has been shown to be related to the incidence of *GV*, while condoms were the least associated contraceptive method demonstrating some level of protection against *GV* infection [15,20]. Natural contraceptive methods rendered an increased incidence of *GV* compared to oral contraception [22]. Insertive methods of contraception, such as copper intrauterine devices (IUDs) and vaginal rings, demonstrated a high level of association between *GV* and vaginal cultures [23-24]. Aside from contraceptives, penetrative toys were also found to be related to a higher likelihood of *GV* colonization. In a cross-sectional study of 320 women, women reporting greater than 10 acts of toy-vaginal sex were 70% more likely to be colonized [18]. 

SES 

Three papers highlighted SES in their assessment of *Gardnerella* prevalence in the population [25-27]. While each of the three papers used their own parameters to define sociodemographic and SES, all three studies found an inverse relationship between SES and *GV* colonization [25-27]. Women with primary school education levels showed higher rates of *GV* colonization compared to those with secondary or university education levels [26,27]. Francis et al. looked solely at Tanzanian girls enrolled in a secondary school with SES being defined by material possessions owned by a household member, with owning a car being the highest status and not owning a cellphone, car, or television being the lowest [25]. Following the pattern, girls deemed as being in the highest SES, with a household member in possession of a car, had the lowest reported incidence of reproductive tract infections [25]. 

These articles not only highlighted the different aspects of SES, but their classifications of each subdivision of SES, such as wealth, were specific to that region and consequently may not be applicable elsewhere [25-27]. 

Ethnicity 

The colonization of *GV* in the vaginal microbiota varies between different ethnic groups. Several studies have been conducted on *GV* prevalence in specific ethnic groups; however, very few have compared their prevalence between ethnic groups. 

Compared to women of Dutch ethnicity, the vaginal microbiota of African Surinamese and Ghanaian (sub-Saharan African descent) women were significantly more likely to contain a polybacterial *GV*-containing VMB [11]. Furthermore, *GV* was the predominant bacteria found in women from sub-Saharan Africa, South Africa, Tanzania, Kenya, and Rwanda [16,25,28-30]. In the USA, women who self-identified as “black” or “African American” had a higher prevalence of *GV* in their VMB compared to women who self-identified as “white” [31-33]. In an Ecuadorian study, *GV* was found in more than 93% of pregnant teenagers [34]. Interestingly, in the context of preterm births, pregnant women who self-identified as “white” were more likely to be colonized by *GV* in their VMB and amniotic fluid compared to pregnant women who self-identified as “black” [32]. 

Smoking 

Multiple studies have been conducted aiming to find a correlation between smoking and an altered VBM, which can increase the predilection for urogenital disease pathology. While the exact pathogenesis for the increase in *GV* species is still unknown, studies have unanimously found a correlation between *GV* and smoking, including both cigarette and marijuana products. These studies have shown a markedly increased presence of *GV* in relation to *Lactobacillus* in smokers versus nonsmokers [14,20,27,32,35-39]. When looking at metabolic profiles, other studies have concluded the breakdown products of the chemicals in cigarette smokes may be the ultimate culprits that lead to the dysregulation of the vaginal microbiota that in turn create a more favorable environment for the overgrowth of “bad” bacteria [35]. In the aforementioned study, nonsmokers were more likely to have a 39-fold increase in *Lactobacillus,* while smokers were more likely to be *Lactobacillus*-depleted [35]. 

While there have been established studies on smoking metabolites, the by-products of marijuana are lesser known. A study conducted with marijuana users found a statistically significant correlation between marijuana use and recurrent BV infections caused primarily by *GV* [37]. 

Genetics 

Few studies have shown that variations in bacterial genetic factors may be associated with the prevalence of *GV* in certain populations. The main gene of interest in studying *GV* is the sialidase A gene, an important virulence factor, which is higher among women with detectable *GV* [25]. 

Anatomical Variations 

A single study analyzed the association of cervix length with the risk of *GV* infection. It was noted that there is a fourfold increase in the abundance of *GV* in women with short cervixes (<25mm) compared to those with longer cervixes (>25mm) [40]. 

Hygiene Practices 

Hygiene practices may also be correlated with the increased incidence of *GV*. One study demonstrated an increased risk of *GV* in women who performed vaginal douching as part of their customary personal hygiene practices [15]. 

**Figure 2:**
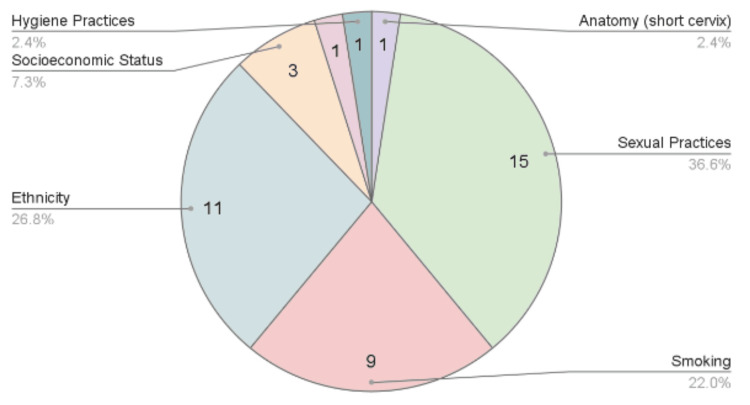
Pie chart of Gardnerella risk factors This pie chart summarizes the findings of the studies recorded in Table 1 and groups the *GV* risk factors based on the number of papers identified in this review [10-40].

Figure 2 shows a pie chart of the most prevalent evidence-based risk factors of *GV* based on the number of papers found in this scoping review of recent literature: sexual practices with 15 papers, followed by ethnicity with 11, smoking with nine, SES with three, and genetics, hygiene practices, and cervix length with one each. 

Table 2 shows all nine risk factors from this study, including contraception use, number of sexual partners in three months, type of intercourse, ethnicity, smoking, SES, hygiene practices, genetics, and anatomical variations. Each risk factor was further divided into point values that represented tier-relative risk profiles inferred from the analysis of the included articles. No contraception use was found to be the most risky for *GV* infections, so it was given a point value of 3, followed by IUDs/vaginal rings given 2, and oral contraceptive pills given 1. Greater than one sexual partner in the past three months was considered riskier and is given a point value of 2, and one or fewer sexual partners were given 1. Penile-vaginal sex was given 3 points, followed by digital-vaginal and penetrative toy-vaginal that were both given 2. Oral-vaginal was considered the least risky, which is given 1 point. Being of African descent was given a risk point value of 5, followed by pregnant women of European or South American descent given 4, pregnant women of African descent given 3, European or South American descent given 2, and finally all other ethnicities given 1. Smoking more than 20 cigarettes per day was given 4 points, 10-20 cigarettes per day was given 3, less than 10 cigarettes per day was given 2, and nonsmokers were given 1. Low SES was given a risk point value of 3, middle SES was given 2, and high SES was given 1. The vaginal douching practice was given a value of 2, while no vaginal douching was given 1. Sialidase A gene-positive individuals were given 2 points, while negative individuals were given 1. Those with cervixes less than 25mm (about 0.98in) were given 2, and those with cervixes greater than or equal to 25mm were given a point value of 1. To determine the value ranges for high, moderate, and low risk, we first calculated the highest and lowest scores possible, which were 33 and 8, respectively. The difference between these total scores was found (25) and subsequently divided into three (8.33). After splitting each risk category into containing around 8 points each, we finally determined that a total sum of all risk factors equivalent to 26 and above was defined as high risk for *GV* infection, a total between 17 and 25 was moderate risk, and a total of 16 and under was considered low risk (Table 3). 

**Table 2:** Risk factor screening tool for Gardnerella infection IUDs: intrauterine devices. This table categorizes *GV* risk factors based on severity. Source: [10-40].

Risk factors for Gardnerella infection		Points
Contraception use	No contraception	3
	IUDs/vaginal rings	2
	Oral contraceptive pills	1
Number of sexual partners in three months	>1	2
	≤1	1
Type of Intercourse	Penile-vaginal	3
	Digital-vaginal	2
	Penetrative toy-vaginal	2
	Oral-vaginal	1
Ethnicity	African descent	5
	European or South American descent, pregnant	4
	African descent, pregnant	3
	European or South American descent	2
	Other	1
Smoking	Heavy (20+ cigarettes/day)	4
	Moderate (10-20 cigarettes/day)	3
	Light (<10 cigarettes/day)	2
	Never	1
Socioeconomic status	Low	3
	Middle	2
	High	1
Hygiene practices	Vaginal douching	2
	No vaginal douching	1
Genetics	Sialidase A gene positive	2
	Sialidase A gene negative	1
Anatomical variation	Short cervix (<25mm)	2
	Long cervix (>25mm)	1

**Table 3:** Screening criteria tool key *GV*: *Gardnerella vaginalis.* This table proposes a point-based system to categorize patients into low risk, moderate risk, and high risk for *GV* infections.

Total	Risk
26-33	High risk for *GV* infection
17-25	Moderate risk for *GV* infection
8-17	Low risk for *GV* infection

Discussion 

BV is known as a condition caused by the disruption of normal vaginal flora. As a result of the overgrowth of *GV* in proportion to the protective, *Lactobacillus* species, the *GV*-to-*Lactobacillus* ratio is increased in BV. BV is commonly misunderstood as a sexually transmitted disease due to its most commonly associated risk factor of risky sexual practices [41]. Given this misunderstanding, public health campaigns can be tailored to improve awareness and education about BV, including the distribution of pamphlets and flyers in obstetrics and gynecology (OB-GYN) and primary care offices, presentations in school health classes, and spreading awareness through social media by medical professionals. Based on the data from this review, risky sexual behaviors include factors such as completion of sexual debut, the presence of multiple sexual partners, lack of condom usage during intercourse, increased penetrative forms of sexual intercourse, insertive sex toys, and vaginal douching [15-21,23,24]. This information suggests that the introduction of foreign materials may lead to mechanical vaginal tissue trauma, disrupting *Lactobacillus* species’ protective defenses of the natural vaginal environment and increasing susceptibility to GV colonization [42]. This mechanism of infection also explains why some data from this review point toward insertive forms of contraception, such as copper IUDs and vaginal rings, leading to an increased risk of *GV* [23,24]. However, since contraception is beneficial for preventing unwanted pregnancies and treating hormonal imbalances, it may be useful for those predisposed to *GV* to consider non-insertive forms of contraception, thereby decreasing the potential for vaginal tissue trauma. Furthermore, considering that vaginal douching is a common hygiene practice with known associations with vaginal infections, it is imperative that physicians educate women to utilize alternative low-risk hygiene practices, such as regular cleaning of the genital area with mild soap and warm water, use of breathable cotton underwear, and avoidance of irritants.

Based on the analysis of the findings, risky sexual behaviors hold a higher risk of *GV* colonization than SES. However, SES is an important factor to consider, as the aforementioned risky sexual practices have shown increased prevalence in populations of lower SES [43]. This is due to reasons such as increased rates of high school dropout , lack of access to resources such as childcare and safe environments, and earlier sexual debut associated with populations of lower SES [44]. Therefore, educational programs targeting these specific populations may help mitigate the incidence of *GV*. This happens by tailoring resources and being both mindful and considerate of the cultural, traditional, and spiritual components of education on sexual practices. In addition, independent of SES, sexual practices may be seen as taboo in certain cultures, potentially leading to a lack of sexual health education and inaccessibility to sexual healthcare [45]. Consequently, it is imperative for healthcare providers to educate both themselves and high-risk patient populations and provide culturally sensitive educational resources and timely access to care. 

Geographic location and ethnicity can alter the microbial composition of the VMB, potentially due to differences in access to healthcare and cultural practices regarding vaginal care, as well as genetic factors. At baseline, black women have a higher probability of having a *GV*-predominant VMB [11]. Additionally, compared to the VMB of women classified as white, the VMB of women classified as black has a significantly higher probability of *GV* colonization after exposure to risk factors [11]. While culturally varied diets and hygiene practices could account for this variability, the possibility of a genetic component cannot be ignored. After controlling for factors that are known to increase *GV* prevalence, such as sociodemographic, sexual risk behaviors, vaginal cleansing practices, and hormonal contraceptive use, women of sub-Saharan African descent were significantly more likely to have a *GV*-containing VMB than Dutch women, who were more likely to have a *Lactobacillus*-dominant VMB, which is commonly accepted as the healthier VMB [11]. Similar results were noted in a study where external factors, such as smoking and multiple sexual partners, were not statistically significant, suggesting variability in the VMB in healthy women among ethnicities [28]. From these results, it can be interpreted that black women have an increased risk of contracting BV or that the definition of BV must be curated differently depending on the ethnic background and genetic makeup of the patient.

The observation of increased *GV* colonization in pregnant women interestingly contradicts trends in nonpregnant populations. One hypothesis that may explain why *GV* prevalence shifts in pregnancy is due to the downregulation of T-helper 1 (Th1)-mediated immunity and upregulation of Th2-mediated immunity during pregnancy to accommodate a growing fetus [1]. The suppression of Th1 responses in pregnancy reduces the ability to control *GV* populations in the VMB [1]. In addition, the Th2-dominant environment in pregnant females may reduce the ability to control *GV* virulence factor, sialidase A, facilitating *GV* colonization [38]. Pregnancy-related hormonal changes may also affect vaginal pH and glycogen content, creating more favorable conditions for *GV* proliferation [38].

The premise of BMI was based on the work of a Belgian astronomer in the 19th century who sampled a group of high-income, mostly white men, which aimed to represent the typical sizes of the total population and determine the “ideal body weight” [44]. This BMI continues to be used in medicine today, even though new studies have shown some people in the “overweight” BMI category, typically mislabeled due to varying ethnic body compositions, have a lower risk of death from heart-related causes than those with a “normal” BMI [44]. This shows that ethnicity must be considered a factor when deciding screening and treatment plans for patients with BV. Changing the way providers view certain risk factors of disease, such as BMI depending on ethnicity, is not a new phenomenon, yet an ongoing development of understanding differences between ethnicities during screening and prior to treatment. Future studies on different ethnicities in different geographical locations should be conducted to determine the specific role and relationship of these two variables on *GV*-to-*Lactobacillus* ratio. 

Since *GV* prevalence has shown to be higher among certain populations of women, the prevalence of an important *GV* virulence factor, sialidase A, among different ethnicities can be investigated [25]. The sialidase A gene codes for sialidase, an enzyme that cleaves sialic acid residues from glycans in the cell wall, such as glycoproteins and glycolipids, which in turn aids in the adherence and colonization of *GV* [25]. This gene is higher among women with detectable *GV* and must be further explored as a potential diagnostic factor when diagnosing BV. In addition, further *GV* virulence factors can be investigated for use in clinical practice to potentially predict future adverse complications in patients. BV-associated *GV* strains demonstrated increased virulence by encoding mucin-degradable protein and biofilm-associated protein genes [46]. While there may be more genes implicated in the contraction of *GV* infection, the lack of research in this sphere renders genetics a low-risk factor compared to sexual practices, SES, and ethnicity. 

Smoking was the second highest risk factor for *GV* infection after sexual practices. Cigarette smoking is a risk factor that has a predisposition toward *GV*-predominant VMBs [35]. Women who reported smoking nicotine products, particularly in the last three months, had an increased risk of *GV* infection [14]. While all studies investigating the relationship between *GV* infections and nicotine by-products from cigarette smoking have unanimously concluded there is a direct relationship between the two, the exact pathophysiology is still unknown [13,20,24,30-33]. Several studies have hypothesized that smoking metabolites may not necessarily be creating an unfavorable microenvironment for beneficial bacteria such as *Lactobacillus*, but rather, they are creating a favorable state for pathogenic bacteria, such as *GV*, to replicate [35,36]. Hippurate, a normal excretory product found in urine, is a well-known substrate utilized by *GV*, and its abundance increases with exposure to the breakdown products of cigarette smokes [35]. Therefore, similar to the use of VMB biomarkers to detect diseases such as high-risk human papillomavirus infections, hippurate concentrations in the urine may be a useful biomarker for assessing the risk of *GV* infections in smokers [47]. 

While there have been established studies on nicotine metabolites, the effects of marijuana use are lesser known. While there was a twofold increase in recurrent BV infections in marijuana users, a metabolic profile was not completed in these patients [33]. Therefore, while it has been established that marijuana affects the VMB similarly to nicotine, there is no definitive data on the pathophysiology by which this is occurring [48]. Future studies should be conducted to determine the breakdown products of marijuana and their respective effects when building up in the vaginal tract or cervical mucus membranes. 

Though low risk, anatomical factors also play a role in *GV* prevalence. The data indicate a higher risk of *GV* infection in women with shorter cervixes, presumably drawing to the hypothesis that a smaller cervicovaginal surface area allows *GV* to overpopulate the VMB [40]. However, the exact mechanism behind increased *GV* prevalence in women with shorter cervixes continues to remain unknown [40]. The abovementioned possible correlation highlights the need for further research to be conducted, to explore a possible direct correlation between variations in anatomical length and GV infection. Furthermore, in regard to age, after further evaluation of all articles chosen for the scoping review, no consensus was reached on the association of age with the presence of *Gardnerella*. 

Regarding limitations, the following paragraph outlines the ones in this scoping review. In regard to SES, the classifications of each subdivision, such as wealth, were specific to the geographic region being studied and consequently may not be applicable in other locations. Therefore, not all factors are as universal as education, and reproducibility utilizing the same criteria in different geographical locations may be challenging. Furthermore, SES and education may differ in their influence and hence importance on *GV* in different geographical locations. In addition, investigating the genetic component of bacterial variations among ethnicities in the VMB is imperative to determine whether or not those variances describe a pathological process or are healthy for a certain population. Furthermore, it is important to note that this review may not include all the subsets of one risk factor. For example, in regard to smoking, there are many other nicotine and non-nicotine alternatives outside of cigarettes and marijuana including but not limited to electronic cigarettes, nicotine patches, vapes, and chewing tobacco. While the mechanism by which these products damage the vaginal and genital tract may be similar, further studies are needed to elucidate the pathogenicity of each when compared against one another. Furthermore, there are confounding variables in many of these studies, such as ethnicity, sexual practices, age, and variances in the VMB with the typical menstrual cycle, and therefore each of the highlighted risk factors cannot be assessed independently. The screening criteria were created with the information acquired through the analysis of this topic; it should act as a template that can be altered ,as further information continues to be understood regarding the risk factors leading to BV. 

## Conclusions

Based on the various risk factors identified in this review, it can be inferred that a patient who presents with a greater number of independent risk factors confers a higher risk of developing a *GV* infection. Timely screening of *GV* is vital, especially in high-risk populations, such as pregnant and immunocompromised patients, who may present with more severe and exaggerated symptoms if they contract BV. Educating physicians and healthcare providers on such risk factors would allow them to better identify these at-risk populations.
